# Spatiotemporal Epidemiology of Tuberculosis in Thailand from 2011 to 2020

**DOI:** 10.3390/biology11050755

**Published:** 2022-05-16

**Authors:** Kawin Chinpong, Kaewklao Thavornwattana, Peerawich Armatrmontree, Peerut Chienwichai, Saranath Lawpoolsri, Udomsak Silachamroon, Richard J. Maude, Chawarat Rotejanaprasert

**Affiliations:** 1 Princess Srisavangavadhana College of Medicine, Chulabhorn Royal Academy, Bangkok 10210, Thailand; kawin.chi@edu.cra.ac.th (K.C.); kaewklao.tha@edu.cra.ac.th (K.T.); peerawich.arm@edu.cra.ac.th (P.A.); peerut.chi@cra.ac.th (P.C.); 2 Department of Computer Engineering, Faculty of Engineering, King Mongkut’s University of technology Thonburi, Bangkok 10140, Thailand; 3 Department of Tropical Hygiene, Faculty of Tropical Medicine, Mahidol University, Bangkok 10400, Thailand; saranath.law@mahidol.ac.th; 4 Department of Clinical Tropical Medicine, Faculty of Tropical Medicine, Mahidol University, Bangkok 10400, Thailand; udomsak.sil@mahidol.ac.th; 5 Mahidol-Oxford Tropical Medicine Research Unit, Faculty of Tropical Medicine, Mahidol University, Bangkok 10400, Thailand; richard@tropmedres.ac; 6 Harvard T.H. Chan School of Public Health, Harvard University, Cambridge, MA 02115, USA; 7 Centre for Tropical Medicine and Global Health, Nuffield Department of Medicine, University of Oxford, New Road, Oxford OX1 1NF, UK; 8 The Open University, Milton Keynes MK7 6AA, UK

**Keywords:** tuberculosis, spatiotemporal, cluster, surveillance, Thailand

## Abstract

**Simple Summary:**

This retrospective study was conducted to describe and analyze spatiotemporal patterns of tuberculosis (TB) incidence at provincial level of Thailand. TB notification data from 2011 to 2020 were obtained from the surveillance reporting system of the Bureau of Epidemiology, Department of Disease Control, Ministry of Public Health. Time series and clustering patterns of the occurrence were assessed by using time series and spatial analyses. The findings indicated temporal trends and clusters of the disease occurred in the study area. More specifically, there was an overall decreasing trend, and significantly high-rate clusters were mostly along the border of neighboring countries.

**Abstract:**

Tuberculosis is a leading cause of infectious disease globally, especially in developing countries. Better knowledge of spatial and temporal patterns of tuberculosis burden is important for effective control programs as well as informing resource and budget allocation. Studies have demonstrated that TB exhibits highly complex dynamics in both spatial and temporal dimensions at different levels. In Thailand, TB research has been primarily focused on surveys and clinical aspects of the disease burden with little attention on spatiotemporal heterogeneity. This study aimed to describe temporal trends and spatial patterns of TB incidence and mortality in Thailand from 2011 to 2020. Monthly TB case and death notification data were aggregated at the provincial level. Age-standardized incidence and mortality were calculated; time series and global and local clustering analyses were performed for the whole country. There was an overall decreasing trend with seasonal peaks in the winter. There was spatial heterogeneity with disease clusters in many regions, especially along international borders, suggesting that population movement and socioeconomic variables might affect the spatiotemporal distribution in Thailand. Understanding the space-time distribution of TB is useful for planning targeted disease control program activities. This is particularly important in low- and middle-income countries including Thailand to help prioritize allocation of limited resources.

## 1. Introduction

Tuberculosis (TB), caused by the bacterium *Mycobacterium tuberculosis* (*Mtb*), is a leading cause of morbidity and mortality globally with approximately 1.4 million deaths and 10 million people developing TB in 2019 [1]. The TB burden is particularly high in low- and middle-income countries (LMICs), with over 95% of deaths and nearly 40% undiagnosed in these regions [2]. Limited accessibility to healthcare and the social stigma of the disease contribute to delays in TB detection and poor treatment adherence [3]. *Mtb* complex pathogens are mainly spread by airborne transmission from infected individuals with active disease. Poor living conditions and overcrowding can perpetuate transmission with faster spread during coughing [4]. This disease is not only a public health concern in LMICs but also leads to financial and social insecurity. The economic burden of TB is substantial due to reduced income, logistics, and medical costs with lost annual income estimated at approximately 58% for individuals and 39% for households [5]. Thailand is considered by the WHO as one of the TB highest-burden countries and TB is ranked as one of the top ten national infectious diseases under the Thai National Disease Surveillance System.

For TB control activities, spatial heterogeneity in the distribution of infection has been an important factor determining the effectiveness of national TB control programs, especially in developing countries [6]. Without proper consideration of spatial heterogeneity in disease burden, control programs can be hindered by failure to implement specific interventions to efficiently manage the local disease situation in different areas; thus, a better understanding of TB spatiotemporal epidemiology can inform planning of effective prevention and control strategies by policymakers [7]. This is particularly helpful in LMICs including Thailand, in which it is necessary to prioritize resource allocation by focusing on regions with the highest burden. Moreover, accomplishing disease control objectives at global or national levels requires good knowledge management including spatial analysis of disease distribution serving as a prelude to control activities and future elimination.

With the development of geographical and temporal analysis methods, these analytical tools have been utilized to investigate TB epidemiology in LMICs. For instance, a time series analysis was built on historical data to predict the future disease burden in China [8,9]. Spatial statistics including spatial filtering and cluster analysis have been used to analyze and visualize the spatial patterns of tuberculosis in Asian and African countries [10,11,12]. These studies demonstrated that TB exhibited highly complex dynamics in both spatial and temporal dimensions at different levels. In Thailand, TB research has been primarily focused on surveys and clinical aspects of disease prevalence with little attention on spatial heterogeneity [13,14]. A few studies performed spatial and temporal analyses for a limited area and/or brief time period. For instance, spatial TB hotspot detection was used in one Thai province [10], while another examined the association of TB with social factors in Thailand during a four year period [14]. Although these investigations have provided valuable insights in understanding the spatial patterns and potential risk factors associated with disease occurrence, previous studies had important limitations, and there is additional scope to more broadly employ spatiotemporal analysis and disease clustering in order to more effectively support disease control policies.

In this regard, it is important to further investigate to have a clearer insight of space-time heterogeneity in the occurrence of TB at small spatial and temporal scales to inform targeted public health responses. Therefore, this research was conducted and analyzed using retrospective surveillance data to describe demographic attributes of people with TB and characterize the temporal pattern of incidence using time series analysis. Furthermore, we also aim to identify spatial clusters of TB using data on monthly TB case notifications for the whole country from national surveillance data for the years 2011 to 2020 in Thailand. The findings from this research were discussed and beneficial in the decision-making process for targeting of public health interventions for more effective disease control.

## 2. Materials and Methods

### 2.1. Study Area and Data Sources

The study was conducted in Thailand, which has a population of approximately 70 million people across 77 provinces and borders Myanmar, Laos, Cambodia, and Malaysia. Thailand has a tropical climate with three seasons: rainy (May–October), winter (October–February), and summer (February–May). The study was planned as a retrospective analysis of reported tuberculosis cases collated from the surveillance reporting system, Bureau of Epidemiology, Department of Disease Control, Ministry of Public Health (MOPH). All the cases were aggregated monthly tuberculosis cases notified during the years 2011 to 2020 at provincial level classified as three types in the surveillance system (pulmonary, meningitis, and tuberculosis in other organs). A TB case was identified using one or more of the following criteria [15,16]: (1) diagnosis of TB by a physician with one of the A15–A19 ICD-10 (the 10th revision of the International Classification of Diseases) codes; (2) positive TB result with one or more of the following laboratory tests—sputum smear, sputum culture, GeneXpert MTB/RIF assay, and line probe assay (LPA); (3) current TB treatment with rifampicin or kanamycin. The surveillance data included demographic information as well as month and province of diagnosis. The geographic coordinates of the province centroids and province boundaries were from the GEO package file in the Database of Global Administrative Areas (GADM), a high-resolution database of country administrative areas. Files for analysis were compiled using R programming language.

### 2.2. Rate Standardization

To study spatiotemporal heterogeneity, it is important to account for population at risk when health outcomes are compared across spatial units since incidence may be influenced by numbers of individuals at risk. This requires standardization of the health outcome to control the population effect. Epidemiological standardization techniques can be applied in either direct or indirect manners over a selected standard population as a common reference for comparison [17]. Using indirect standardization, the ratio of observed to expected health outcomes is calculated as a standardized ratio. The standardization can also help to decrease the influence of areal effects due to small numbers of the outcome, which could lead to an unstable rate and large errors of estimation [18,19,20]. The standardized mortality or incidence ratio for province i at time period t,$\;x_{it}$, was calculated as the ratio between incidence or death count per population size [21] as *x_it_* = *N_it_*/*P_it_*, where $N_{it}$ is the TB incidence or death counts and $P_{it}$ is the number of population at risk.

### 2.3. Descriptive and Temporal Analyses

TB cases were analyzed at provincial level and aggregated by age group for descriptive analysis. The demographic data were analyzed using R version 1.3.1093. Temporal patterns were assessed using the monthly numbers of cases and analyzed by the seasonal-trend decomposition procedure based on the Loess smoother (STL) [22]. The TB time series data were extracted into components *Y_t_* = *m_t_* + *s_t_* + *e_t_*, where $Y_{t}=\sum_{i}x_{it}$ represents the incidence or death count of TB combined from all provinces, $m_{t}$ is the trend component, $s_{t}$ is the seasonal, and $e_{t}$ is remainder or residual at province *i* and month t [23,24]. The procedure starts from trend calculation using a moving average method as ${\hat{m}}_{t}=\frac{1}{2k+1}\;\sum_{j=-k}^{k}Y_{t+j\;}$, where ${\hat{m}}_{t}$ is the estimate of the trend-cycle at time t obtained from the averaged number of events of the time series within k months of t (set iterator $j\in \left[-k,k\right]$). Then, the seasonal component, $s_{t}$, can be computed by evaluating seasonal index for each period, which is averaged over the detrend values for the same month for all periods while the residuals, $e_{t}$, are calculated using differencing from all known variables. The remainder is the residual left in the time series data after removing its trend, cycle, and seasonal components. This component is the random fluctuation in the time series data that the other components in temporal decomposition cannot explain. In this study, we performed the temporal analysis on the TB data over the 10-year period using forecast version 8.14 and fpp2 version 2.24 (Hyndman, Australia) libraries in R version 4.1.0.

### 2.4. Spatial Pattern Analysis

#### 2.4.1. Spatial Contiguity Matrices

For spatial pattern analysis in this study, we focused on spatial cluster detection. In the following sections, we first describe the spatial contiguity matrix for the neighboring structure we used. Then, global autocorrelation testing was explained and used to assess the overall clustering property in the data. Finally, local hotspot detection was applied to appropriately identify specific cluster locations.

**Figure 1:**
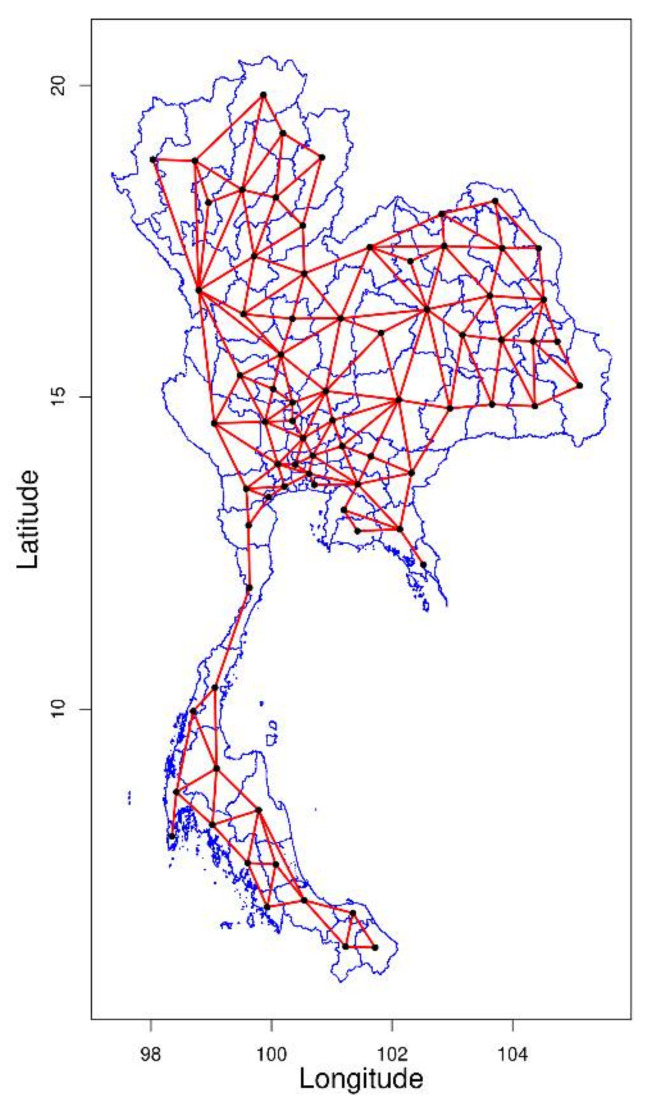
Map of Thailand with provincial borders (blue lines) and connected provinces from spatial contiguity matrix (black dots and red lines).

In the first step to identify the neighborhood contingency, a spatial contiguity or spatial weight matrix, W, is a square matrix that defines spatial contiguity for all node pairs, with a zero diagonal, and off-diagonal nonzero elements. This matrix is required for spatial autocorrelation calculation between the value of a provincial node and its neighbors that appear in global and local detection. A general approach to specify the matrix is to calculate the distance band labeling the node-pair compared with a distance threshold. However, for the areal data in our study, which does not include patient coordinates, the boundary-based contiguity matrix might be more appropriate. There are four main types of contiguity matrix associated with the game of chess: linear, rook, bishop, and queen contiguity, perhaps the most widely used due to the flexibility to capture various autocorrelation forms. The main concept of the queen contiguity criterion is considering adjacent neighbors mutually sharing borders and edges. For each province-pair $\left(i,j\right)$ in our study map, the members $w_{ij}$ and $w_{ji}$ in the queen contiguity matrix W were set to 1 when *i* = *j*, otherwise as 0. The adjacent matrix was processed from the Thailand provincial-level geopackage file (gkpg extension, retrieved from GADM) loaded using GeoPandas 0.9.0 (Jordahl et.al.) and calculated queen contiguity matrix using libpysal 4.4.0 (Rey and Anselin) [25,26] as represented in Figure 1.

#### 2.4.2. Global Spatial Detection

Global detection was applied to identify the overall clustering property in the data. In general, global cluster tests are used to examine whether autocorrelation exists in the dataset without being concerned with the specific coordination of clustering [27]. There are several approaches for global detection, e.g., Geary’s c statistic that relies on sum squared differences of adjacent pairs of values, and Moran’s I coefficient that considers spatial patterns compared with the random form. Despite the various applications of Geary’s c and Moran’s I, these methods do not test under hotspot and coldspot segregation [28]. On the other hand, the Getis-Ord G statistic considering the summation of values associated with adjacent points might be more suitable for our study.

In general, the global Getis-Ord statistic, G, is used to measure the overall spatial association of values that have polygon contiguity [29,30]. As the general case is that no particular location i is fixed, in the global detection case, the statistic equation can be expressed as

(1) $$G=\frac{\sum_{i=1}^{n}\sum_{j=1}^{n}w_{ij}x_{i}x_{j}}{\sum_{i=1}^{n}\sum_{j=1}^{n}x_{i}x_{j}},\;j\neq i\;$$

where $w_{ij}$ is the spatial weight value in the spatial matrix, and $x_{i}$ is the standardized rate for province *i*. The normalized G (Z-score, $Z_{G}$) score was used for hypothesis testing under the null hypothesis of spatial independence. Highly positive $Z_{G}$ values indicate clustering of areas with similarly high health outcomes, whereas highly negative $Z_{G}$ values indicate the tendency to have similarly low health outcomes. The significance of the global Getis-Ord statistic was then evaluated under permutation testing distribution using esda 2.3.6 that is a subset of the libpysal library 4.4.0 (Rey and Anselin) in Python (Rossum, Drake, and Fred, CA) [26].

#### 2.4.3. Local Spatial Detection

Due to the limitation of global detection only concerning the overall clustering property, local detection was also applied to appropriately identify specific cluster locations [31]. Local Moran’s I and local Geary’s C are common measures that only calculate the similarity with neighbors and classify clusters into outliers. To inform spatial locations for effective public health control planning, the hotspot analysis calculating high–low values for cluster detection such as local Getis–Ord $G_{i}^{*}$ statistic would be more appropriate for the purpose of our study.

The local Getis–Ord statistic, $G_{i}^{*}$, compares local averages for each province (province itself included) to global averages from overall area to identify high-value and low-value clusters. For province i, the standardized Getis–Ord $G_{i}^{*}$ in the distribution of the population density form can be defined as [11,32]

(2) $$G_{i}^{*}=\frac{\sum_{j}w_{ij}x_{j}-W_{i}\bar{X}}{\sqrt{\frac{\left(nS_{li}^{*}\right)-W_{i}^{2}}{n-1}}},\;\forall j$$

where W is a spatial matrix that includes self-loop for all provinces (set $w_{ii}=1$ for all i), $w_{ij}$ is the spatial weight value in spatial matrix W, $x_{j}$ is either standardized incidence or mortality rate for province j, $W_{i}$ is the sum of value from matrix W in position i, $S_{li}^{*}$ is sum square weight only row i, and $\bar{X}$ and s denote mean and variance for all n provinces. This calculation relies on the same hypothesis as global detection but considers contiguous neighbors of province i instead. Provinces that contain an extremely high $G_{i}^{*}$ value were considered as part of a high-value cluster (hotspot cluster) and those with an excessively low $G_{i}^{*}$ value were assigned to a low-value cluster (coldspot cluster). The significance of $G_{i}^{*}$ statistic was tested under the permutation distribution as for global detection. The detected significant hotspots/coldspots imply that high/low TB cases in a particular province were surrounded by others with high/low TB cases, respectively. To classify a province as a hotspot, a threshold Z-score of >1.96 equivalent to the level of significance at 0.05 was considered. Others with Z-scores between −1.96 and +1.96 were considered as having nonsignificant clusters while those with Z-scores < −1.96 were classified as coldspots. The Getis–Ord $G_{i}^{*}$ statistic was computed for provincial standardized mortality and incidence rates using library esda 2.3.6 (Rey and Anselin) with geopandas 0.9.0 (Jordahl et al.) and matplotlib 3.4.2 (Hunter, UT) for spatial visualizations [26,33].

## 3. Results

This section may be divided by subheadings. It should provide a concise and precise description of the experimental results, their interpretation, as well as the experimental conclusions that can be drawn.

### 3.1. Descriptive and Temporal Analyses

**Figure 2:**
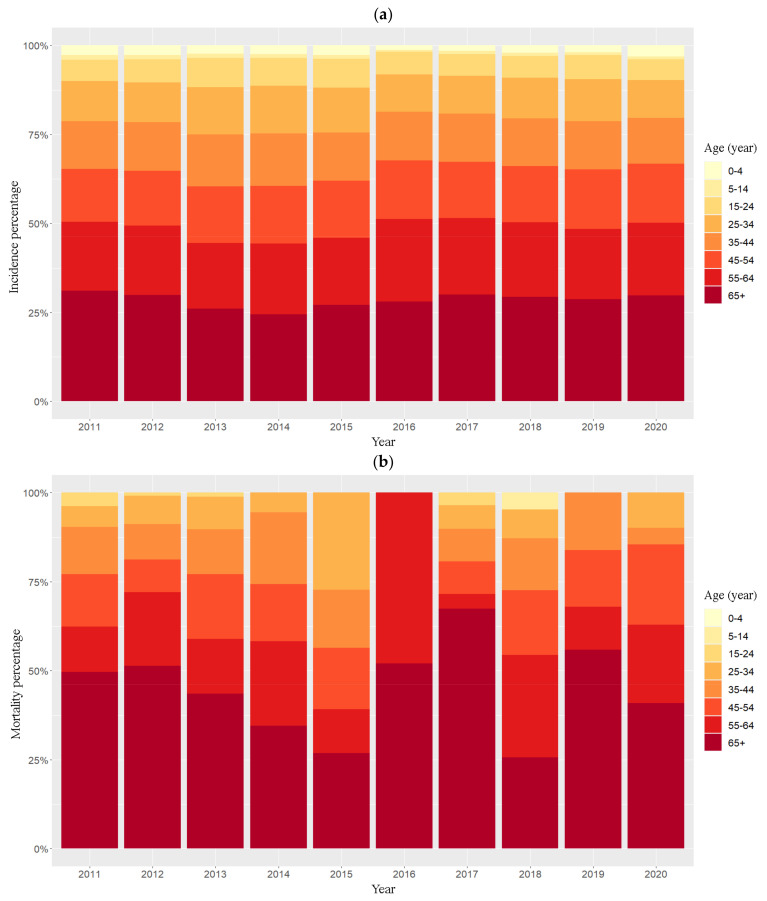
Annual proportion of standardized incidence (**a**) and mortality (**b**) by age group during 2011–2020.

In total, cases and deaths notified in the national surveillance system during 2011 to 2020 were 143,997 and 400, respectively, as shown in Table 1. Pulmonary TB accounted for 67.91% of cases and 89.3% of deaths, the remainder being extrapulmonary (meningitis and other organs), as shown in Table 1. Annual proportions of standardized incidence and mortality are shown in Figure 2. People over 65 years old comprised 28.46% of standardized incidence and 44.78% of standardized mortality overall, followed by those aged 55–64 years with 20.16% of standardized incidence and 20.01% of mortality. Both incidence and mortality rate decreased with decreasing age but age distributions did not change over time. The total proportions in children less than 15 years old were 3.26% of standardized incidence and 0.48% of mortality while there were no deaths for children 0–4 years old.

**Figure 3:**
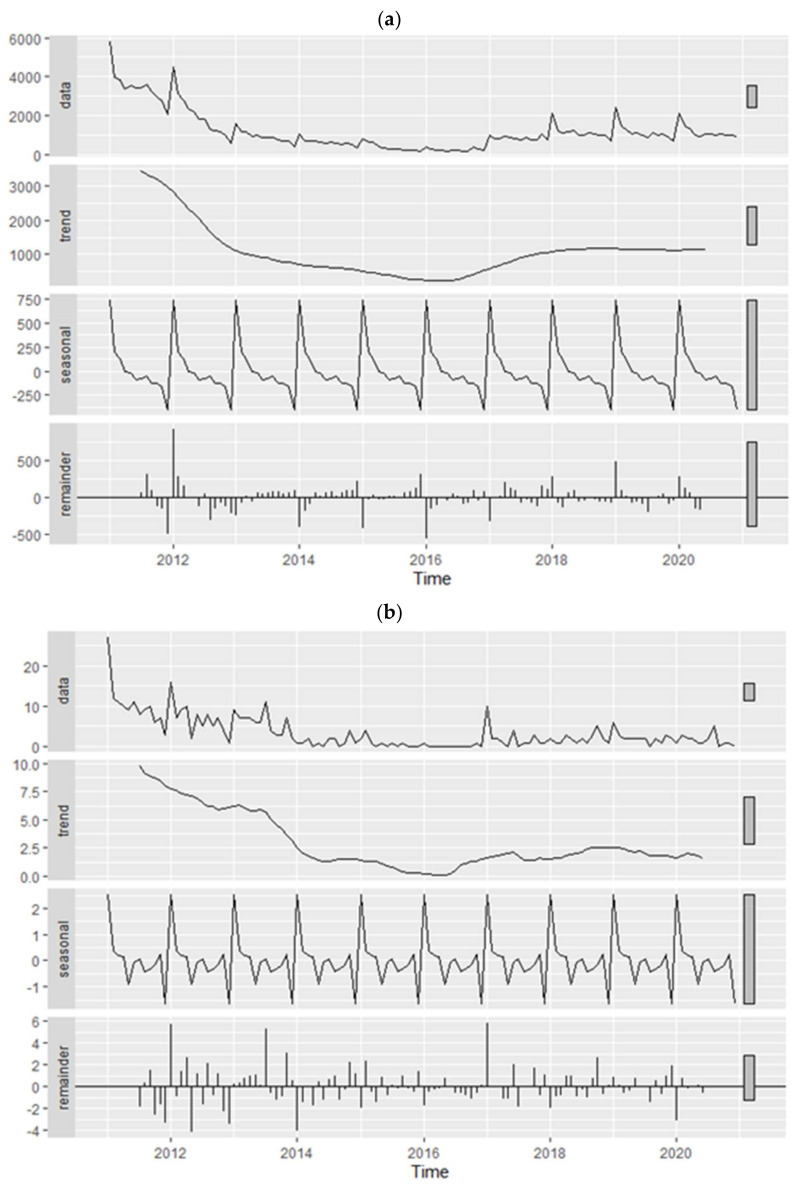
Time series decomposition of the monthly notified TB incidence (**a**) and mortality (**b**) during 2011–2020.

Figure 3 shows the time series decomposition of the TB cases (Figure 3a) and deaths (Figure 3b) notification data. The incidence rates tended to decrease from 2012 to 2016 and increased in 2017 followed by a levelling off, as seen from the trend component. For the seasonal variation, both incidence and mortality peaked early in each calendar year and decreased with fluctuation toward the end of the year.

### 3.2. Spatial Pattern Analysis

#### 3.2.1. Global Spatial Detection

The general G statistics were applied to examine the global autocorrelation in each time period to evaluate the spatial correlation over the study areas. Since this measure only tests the overall pattern of spatial pattern against randomness and could miss isolated hotspots if only strong global autocorrelation is considered, the level of significance was relaxed to 0.1 to initially assess the global spatial behavior. The results of global G statistics suggested spatial autocorrelation in both incidence and mortality. It was found that the test values for incidence rate were statistically significant (*p*-value < 0.1) only in 2012 and 2013 while the mortality rates were found to be spatially clustered only in 2011 to 2013 and 2015 to 2016, as presented in Table 2.

#### 3.2.2. Local Spatial Detection

**Figure 4:**
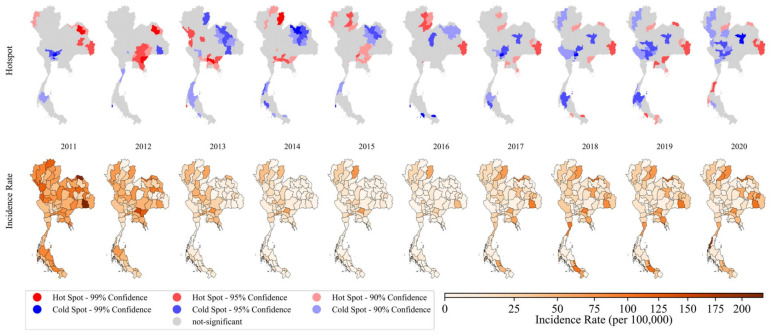
Maps of yearly provincial incidence clusters using local Getis–Ord statistic (upper) and crude TB incidence rates (lower) during 2011–2020.

To further investigate the local behavior, a more specific detection using the Getis-Ord $G_{i}^{*}$ statistic was performed for each province for all study periods. In Figure 4, hot (red) and cold (blue) spot incidence maps are shown in the top panel with standardized incidence rates of provincial TB in the bottom panel. The incidence decreased from 2011 through 2016, then increased in 2017 after which they were stable until 2020, the same pattern as was found in the time series analysis. The high-value incidence clusters (hotspot group) were mainly focused along the borders with neighboring countries over the study period. In the east, the hotspots mostly appeared around the provinces of Chachoengsao, Sa Kaeo, and Prachin Buri. In the northeast, bordering Cambodia, the results implied the potential clusters were in Ubon Ratchathani, Si Sa Ket, Yasothon, and Amnat Charoen while other high incidence zones in the northeast were suggested to be Sakon Nakhon, Nakhon Phanom, and Bueng Kan. In northern areas, hotspots included Chiang Rai, Lampang, Nan, Phayao, and Phrae during 2015–2020 while there were other foci in the west including Tak, Kamphaeng Phet, and Nakhon Sawan.

**Figure 5:**
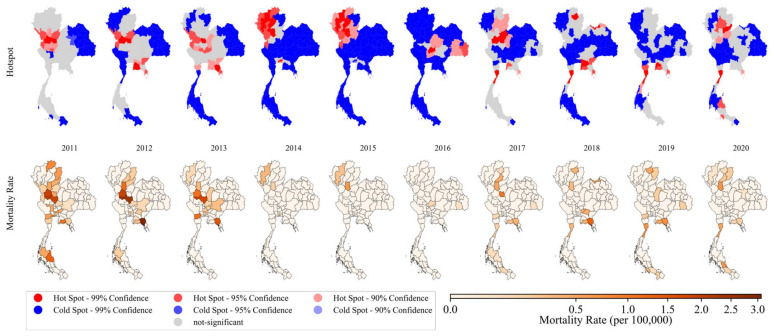
Maps of yearly mortality clusters using local Getis–Ord statistic (upper) and crude TB mortality rates (lower) during 2011–2020.

Figure 5 depicts hotspots of TB mortality rates in the top panel and spatial distribution of TB mortality rates in the bottom panel. Provincial mortality rates generally reduced from 2011 to 2016 and slightly increased from 2017 until the end of the study period in 2020, similar to the findings of the time series analysis. For local detection, cluster maps mostly showed coldspots, especially in the northeastern and southern regions. During 2011–2013, hotspots mostly appeared in the western provinces such as Tak, Nakhon Sawan, Kamphaeng Phet, and Phichit. During 2014–2015, the hotspots were mostly in the northern region; then, they were clustered in the provinces along the Thai-Lao border in the regions of Nan, Phrae, and Phayao during 2017–2020. Some provinces in the east and south were also detected as potential hotspots in 2017–2020.

## 4. Discussion

A retrospective analysis was applied to TB surveillance data in Thailand from 2010 to 2020 with age stratification to examine temporal and spatial distributions. In our time series analysis, there was a seasonal pattern in the TB incidence and mortality with an annual peak in winter. A seasonal effect on transmission of tuberculosis has been observed in Peru and China [34,35]. In addition, variations in vitamin D levels, indoor activities, PM2.5 concentration, and seasonal immune function changes could be possible drivers of seasonal tuberculosis [36,37]. In Thailand, high levels of air pollution usually occur during the first quarter of each year, which is the dry season of the country [38], suggesting that air pollution could irritate the respiratory system and trigger the seasonal pattern.

Based on the national operational plan 2017–2021, a detailed analysis of the available data suggested that the disease burden was falling during 2011–2016 due to the advent of the universal health coverage policy; however, there still might be a fraction underreported [39]. The report from the 5th Joint International Monitoring Mission for TB Control (JIMM) suggested that Thailand, compared with other countries in the region, had been facing a number of challenges including under-reporting from non-MOPH health facilities [40], which might have resulted in the decreasing trends of incidence and mortality during 2011–2016. Nonetheless, according to the Thailand Operational Plan to End Tuberculosis 2017–2021, the first strategy was to ensure that all presumptive TB cases have access to TB screening and early TB diagnosis via molecular diagnostics, and to ascertain an effective TB surveillance system, especially in key target populations [39]. So, this suggests an increase in the access to early diagnosis and more TB case findings in key target populations, which might imply the increasing incidence trend after 2016.

In Thailand, the EPTB proportions to the total TB cases were 29% in 2011, which then increased to 36% in 2019. Globally, this TB type represented 15% of the 6.3 million new TB cases reported in 2016, varying from 8% notified in the WHO Western Pacific Region to 24% reported in the WHO Eastern Mediterranean Region [41]. In North America, Sub-Saharan Africa, and Australia, there were 30% of all new TB cases and relapses in 2016 [41]. EPTB rates were different according to the socioeconomic level of the country and to the supports and resources of TB programs. Despite the data reports, studies conducted on extrapulmonary TB have been mostly in high-income countries; thus, there is a need to better understand EPTB in low- and middle-income countries [42]. Moreover, this type of TB is likely to be undiagnosed in developing countries due to the difficulties in its diagnostic process with limited infrastructure. Thus, EPTB deserves greater attention in those areas, especially in resource-limited settings.

Age-standardized incidence and mortality rates were higher with increasing age but the age distribution did not change over time. This is consistent with TB survey estimates using WHO data in African countries [43]. Moreover, a study in the US reported that the estimated latent TB infection prevalence was highest in persons aged >65 years old among the non-US born and highest in persons aged 45−64 years among the US born. The elderly also had the worst outcomes among all the age groups in a study in Zimbabwe, which might be related to immunosuppressant comorbidities or other age-related diseases [44]. TB treatment among senior populations has been found to be complicated by the treatment of comorbidities such as diabetes mellitus, leading to increased adverse treatment effects, mortality, high rates of recurrent TB, and drug resistance [45,46]. Hence, there should be focused attention on the more vulnerable older population with underlying diseases, who have worse treatment outcomes than other age groups.

Incidence rates of notified TB cases exhibited spatial heterogeneity, as indicated by the occurrence of spatial clusters and isolated hotspots in particular areas. This study indicated that statistically significant hotspots and spatial clusters were common along the borders with neighboring countries. From data reported to the WHO, countries neighboring Thailand are among the list of those with a high burden of TB [47]. Tuberculosis infection among migrant workers, especially along the borders, has been one of the major public health problems for Thailand’s TB control program [48,49], as also shown in the hotspot analysis. On the other hand, high-mortality hotspots were mostly in the middle of the country. This difference might be due to the population structure in those areas. Incidence cases along the borders were in areas with high population movement and migrant workers who were of working age, while the central region was more conservative with a more senior population who might also have had underlying health conditions. Nonetheless, further investigation is needed in order to form proper public health interventions.

The TB pattern in the Thai borderland is seemingly related to the mobility of migrants seeking economic opportunities who traversed the borders and traveled across the country for treatment [49]. Some migrants travel from their hometowns to Thailand due to a lack of available, affordable, and appropriate care in those settings [48]. Traveling can be costly and logistically challenging for such migrants, who have often exhausted other options for accessing medical treatment and supportive care. Some have to travel with active TB either to access treatment or to return home to their family [50]. Moreover, legal status and financial difficulties are also related to the social, legal, and economic environments where migrants live [50]. There are also barriers to accessing TB care for migrant populations, including legal and health insurance issues [51]. Therefore, further investigation on migrants is an important issue for TB control activities of the country. In addition, effective disease surveillance and responses are essential and should cover various migrant conditions.

The utilization of spatiotemporal analysis to examine the pattern of TB cases can be beneficial in understanding the space-time distribution and aggregation of TB clusters [7]. Consequently, the results suggest that TB transmission is highly heterogeneous and there may be benefit in using this to guide variation in the intensity of control activities between different administrative units. Areas with high disease rates might require more attention and appropriate support compared to regions with lower risk [52]. Regions with hotspots and clusters of TB notifications should be prioritized for targeted strategies to control TB transmission in those areas. Thus, policymakers and health authorities would need to effectively manage resource allocation for better results from TB prevention and control activities.

Our study had several limitations. It was conducted using a retrospective design with secondary surveillance data; thus, the numbers are heavily dependent on reporting rates. As no data were available on testing, it was assumed that the case numbers reflected a similar proportion of true incidence in all provinces. The TB notifications were aggregated in groups rather than as individuals, which meant the complicated interactions of factors at an individual level could not be examined. Nonetheless, our analyses utilized the data potential by using various analytical tools to investigate both space and time dimensions to more comprehensively inform surveillance activity planning. In addition, we applied a long-period of 10 years (120 months) to examine the disease patterns. Although there are other spatial detection methods such as SatScan [53], which is widely used in spatial analysis, the scan statistics for this are assumed to be circular and, given the irregular shape of the administrative boundaries at provincial level in this study, this could potentially miss hotspots and isolated areas with excess risks. In future work, other spatiotemporal analytical methods such as Bayesian disease mapping could be employed and compared to further examine the spatiotemporal heterogeneity of the disease distribution. Additionally, a focus on the association between TB occurrence and various environmental and socioeconomic risk factors with underlying diseases might improve the knowledge of the disease pattern and better inform control efforts.

## 5. Conclusions

This study utilized spatiotemporal methods to analyze and provide a better understanding of the spatial and temporal patterns of notified TB cases and deaths. The temporal pattern was examined using time series decomposition, and global and local spatial cluster measures were used to reveal geographical characteristics of the disease. The results of the study showed an overall decreasing trend with seasonal peaks in winter. There was observed spatial heterogeneity and disease clusters in many regions, especially along country borders. In Thailand, the TB surveillance system has traditionally been operated passively at health facilities where patients with active TB symptoms seek medical care and healthcare workers can diagnose and provide treatment. However, this surveillance system may be inaccurate to measure the total burden of TB, especially in the remote communities and migrant populations. Our findings can be beneficial in providing more details of the space-time distribution of TB and targeting high-risk populations, which is helpful for effective planning allocation of limited resources for control program activities. It is hoped that our findings can support public health interventions and provide a more comprehensive picture of TB for more effective disease control activities.

## Figures and Tables

**Table 1:** Annual TB cases and deaths by type of infection notified in the surveillance system during 2011–2020.

Year	Pulmonary		Meningitis		Other Organs		Overall	
	Incidence	Mortality	Incidence	Mortality	Incidence	Mortality	Incidence	Mortality
2011	29,825	103	829	6	11,384	14	42,038	123
2012	16,403	75	442	3	7050	4	23,895	82
2013	7630	63	233	6	3225	3	11,088	72
2014	5148	15	154	0	2194	0	7496	15
2015	2958	10	98	0	1572	0	4628	10
2016	1815	1	47	0	1152	1	3014	2
2017	6993	24	150	1	3123	1	10,266	26
2018	8954	22	217	1	4551	1	13,722	24
2019	8839	25	224	1	4823	1	13,886	27
2020	9222	19	231	0	4511	0	13,964	19

**Table 2:** Annual general G statistics for the notified TB incidence and mortality rates in Thailand at provincial level in 2011–2020.

Years	Incidence Rate			Mortality Rate		
	G	*p*-Value	Interpretation	G	*p*-Value	Interpretation
2011	0.0772	0.4087	Scattered	0.2137	0.01	Clustered
2012	0.0913	0.0538	Clustered	0.4168	0.0416	Clustered
2013	0.1096	0.0692	Clustered	0.3245	0.0907	Clustered
2014	0.1193	0.1777	Scattered	0.6526	0.2942	Scattered
2015	0.1246	0.4187	Scattered	1.2458	0.0364	Clustered
2016	0.1331	0.4124	Scattered	1.2255	0.0234	Clustered
2017	0.0875	0.2304	Scattered	0.322	0.3345	Scattered
2018	0.0923	0.4539	Scattered	0.2734	0.2839	Scattered
2019	0.1011	0.301	Scattered	0.2486	0.2691	Scattered
2020	0.0981	0.4566	Scattered	0.2652	0.1931	Scattered

## Data Availability

The datasets analyzed during the current study are available on the surveillance reporting system website, Bureau of Epidemiology, Department of Disease Control, Ministry of Public Health.
